# Nonlinear evolution characteristics and seepage mechanical model of fluids in broken rock mass based on the bifurcation theory

**DOI:** 10.1038/s41598-024-61968-6

**Published:** 2024-05-14

**Authors:** Jia Yunlong, Cao Zhengzheng, Li Zhenhua, Du Feng, Huang Cunhan, Lin Haixiao, Wang Wenqiang, Zhai Minglei

**Affiliations:** 1 International Joint Research Laboratory of Henan Province for Underground Space Development and Disaster Prevention, School of Civil Engineering, Henan Polytechnic University, Jiaozuo, 454000 Henan China; 2 Henan Mine Water Disaster Prevention and Control and Water Resources Utilization Engineering Technology Research Center, Henan Polytechnic University, Jiaozuo, 454000 Henan China; 3 Collaborative Innovation Center of Coal Work Safety and Clean High Efficiency Utilization, Jiaozuo, 454000 Henan China

**Keywords:** Fault water inrush, Nonlinear seepage characteristics, Bifurcation theory, Numerical simulation, Coal, Civil engineering

## Abstract

With the deep extension of coal mining in China, fault water inrush has become one of the major disasters threatening the safety production of coal mine. Based on the control equations of steady state and non-Darcy seepage in fractured rock mass, the multi-parameter nonlinear dynamic seepage equations of fractured rock mass are established in this paper. Based on the nonlinear dynamics theory, the function of the state variable in the system is derived, and the influence of the gradual change of non-Darcy flow factors on the structural stability of seepage system is studied. The research achievements show that there are three branches in the equilibrium state of the seepage system. Specifically, the stability of the equilibrium state changes abruptly near the limit parameter. The seepage dynamic system of fractured rock mass has the delayed bifurcation, and the coal mine disaster such as fault water inrush occurs easily at the bifurcation point. The research results are of great significance to enrich the theory of fault water inrush in coal mine, and to reveal the disastrous mechanism of fault water inrush and guide its prevention and control technology in coal mine, which can provide the theoretical reference for predicting the water seepage stability in fractured rock mass.

## Introduction

In engineering practice, the fractured rock mass of dam foundation, slope and roadbed is often unstable due to seepage instability^1^. After the dam is loaded with water, the upstream fault is stretched and the downstream fault is compressed, forcing the seepage pressure under the dam foundation to rise^2,3^. The seepage pressure water extrudes the fractured rock mass to destroy the dam foundation, and the reservoir water thoroughly erodes the fractured rock mass of the dam foundation, resulting in a series of catastrophic accidents^4,5^. The existence of groundwater in fractured rock mass strongly affects the deformation and stability of rock mass structure through various physical and chemical actions such as lubrication, subduction, hydrolysis, bonding, freeze–thaw, and hydraulic fracturing mechanics caused by hydro-static pressure and dynamic water pressure^6^.

Fractured rock mass is a common deformable porous medium in engineering, which can be roughly divided into two categories^7^. Specifically, one is the in-situ fractured rock mass broken by structural and mining stress but still in the original position (such as the loose ring rock mass around the excavation of the roadway), while the other is the accumulated fractured rock mass broken by engineering excavation and can be compacted again under high pressure (such as the overburden accumulation body broken by mining action). Compared with intact and dense rock mass, fractured rock mass has higher compactibility and permeability, and its permeability coefficient is one to several orders higher than that of intact rock mass. Therefore, the research work of fractured rock mass seepage characteristics has a broad engineering application prospect.

The seepage law of liquid in fractured rock mass has been studied by many scholars. Ma et al.^8^ studied the influence of stress and grade pair on pore size distribution, pore fractal and nonlinear seepage characteristics behavior of fractured rock mass, and built a permeability prediction model of fractured rock mass based on nuclear magnetic resonance. On the basis of collecting and sorting out relevant data at home and abroad, Yang et al.^9^ summarized the research status of nonlinear seepage characteristics water inrush in fractured rock mass from the aspects of nonlinear seepage characteristics theory equation, non-Darcy seepage test and nonlinear seepage characteristics numerical model method. Xu et al.^10^ used the constant head steady state permeability method to study the permeability performance of fractured rock mass in the caving zone of goaf and the permeability coefficient of fractured rock mass with different porosity. The results show that the underground water flow in the fractured rock mass in the caving zone of goaf is characterized by high-speed nonlinear seepage characteristics, and the characteristics of underground water flow are obviously affected by the porosity and particle size distribution of the permeating medium. Zhang et al.^11^ studied the seepage characteristics of fractured rock mass under the confining conditions, and the results show that the porosity of both the collapse column and limestone samples decrease exponentially with the increase of axial pressure, but the porosity of the collapse column samples decrease slightly more than that of limestone. An et al.^12^ analyzed the fault erosion water inrush process under the action of confined water on the floor in detail through numerical simulation. Based on the fractal theory of porous media and the nonlinear seepage characteristics theory, Liu et al.^13^ established a nonlinear seepage characteristics mathematical model of fractured rock mass considering the composition of clay and the effect of mud filling. The results show that with the decrease of the permeability of rock mass in the fractured zone, the critical pressure gradient at the beginning of nonlinear occurrence also increases, and the nonlinear critical pressure gradient can be used to quantify the water-blocking ability of fractured rock mass. Based on the theory of two-phase flow, Du et al.^14^ analyzed the characteristics of water–sand two-phase seepage in fractured rock mass and the applicable theoretical model, and the results show that as the particle size of fractured rock mass increases, the *β* factor and acceleration coefficient of water–sand two-phase non-Darcy flow decreases, and the mobility increases. Xu et al.^15^ carried out a non-Darcy seepage test of fractured rock mass under the action of high hydraulic gradient, and the results show that there is a negative exponential relationship between the non-Darcy equivalent permeability coefficient and the hydraulic gradient, and its value is affected by the pore structure of the sample. Yao et al.^16,17^ conducted a variable mass permeability test of fractured rock mass with different proportions. The results show that the filling content has an important effect on the permeability of fractured rock mass. With the increase of the proportion of fill in the sample, the maximum mass loss rate of the sample increases and the porosity increases. Both the initial permeability and the increase of permeability show a change law of first increasing and then decreasing. Chen et al.^18^ established a method to extract the permeability parameters of fractured rock mass with variable mass based on the time series of pressure gradient and seepage velocity, and analyzed the feasibility and accuracy of this method for calculating the permeability parameters of fractured rock mass with variable mass through a numerical example. Zhang et al.^19^ studied the stress and seepage coupling model of fractured rock mass, and the results show that the increase of pore water pressure reduced the peak strength of rock mass, and the existence of high-pressure water increases the deterioration degree of rock mass and the development degree of cracks, thus increasing the risk of disaster caused by water inrush.

The mechanism of seepage disaster in fractured rock mass has become an important research topic in the mechanism of water inrush^20^. In summary, there are three main hypotheses to explain the mechanism of water inrush in coal mines, which are water inrush hypothesis caused by rock mass structure failure, water inrush hypothesis caused by seepage and loss stability, and water inrush hypothesis caused by rock mass deformation and seepage instability in coupling system. The hypothesis of seepage instability is that the accidents of water inrush, gas outburst and sand collapse in coal mine are the manifestations of seepage instability. Li et al. ^21,22^ established a dynamic model of non-Darcy seepage flow in fractured rock mass, without involving the mass change. For such fractured rock mass that does not consider the mass change in the process of infiltration, the key point to explain the mechanism of water inrush from the perspective of seepage instability is whether the instability condition of the seepage system can be physically achieved. Li et al.^23^ studied and discussed whether the instability condition can be physically achieved. Bifurcation theory is widely used. For example, a non-smooth bifurcation exists in a grid-connected inverter controlled by a generator^24^. In terms of turning direction stability of high-speed UAV, saddle knot bifurcation and Hopf bifurcation occurs in the system^25^. In this paper, the structural stability of seepage system in fractured rock mass is studied by numerical response analysis based on bifurcation theory and nonlinear seepage dynamics equation.

## Control equation of nonlinear seepage characteristics

### One-dimensional nonlinear seepage characteristics dynamics equation

The governing equation of nonlinear seepage characteristics in fractured rock mass consists of three parts, namely, the mass conservation equation, the seepage motion equation and the state equation^26^.

1. When fluid flows in porous media, it follows the law of conservation of mass, and the differential equation satisfied by this conservation of mass is the continuity equation:
  1 $$ \overline{\nabla } \cdot (\rho v) + \frac{\partial (\rho \phi )}{{\partial t}} = \rho q $$
  where *v* is the seepage velocity, $\phi$ is the porosity, $\rho$ is the mass density of the fluid, and *q* is the source term.

For passive unsteady seepage, the continuity equation can be simplified as follows:

2 $$ \overline{\nabla } \cdot (\rho v) + \frac{\partial (\rho \phi )}{{\partial t}} = 0 $$

For one-dimensional unsteady flow, $v_{x} = v$. Applying the continuity equation, we can get

3 $$ \frac{\partial (\rho \phi )}{{\partial t}} + \frac{\partial (\rho v)}{{\partial x}} = 0 $$

(2) Darcy’s law is the motion equation of steady state seepage flow. For more general unsteady Darcy seepage flow, the following motion equation is given in the literature^27^:
  4 $$ \rho g_{a} \frac{\partial v}{{\partial t}} = - \nabla p - \frac{\mu }{k}v + \rho g $$
  where $g_{a}$ is called the acceleration coefficient tensor. Permeability *k* is a scalar for homogeneous isotropic media and a second-order tensor for anisotropic media.

For unsteady nonlinear seepage characteristics flow of fractured rock mass, its one-dimensional motion equation can be expressed as:

5 $$ \rho g_{a} \frac{\partial v}{{\partial t}} = - \frac{\partial p}{{\partial x}} - \frac{\mu }{k}v - fv^{2} - \rho g $$

where $g_{a}$ is the acceleration coefficient, *f* is the Darcy flow deviation factor, *p* is the pore fluid pressure, *μ* is the dynamic viscosity of the fluid, and *k* is the permeability of the broken rock.

(3) The equation of state is^28,29^:
  6 $$ \rho = \rho_{0} [1 + c_{f} (p - p_{0} )] $$
  7 $$ \phi = \phi_{0} [1 + c_{\phi } (p - p_{0} )] $$
  where $\rho_{0}$ and $\phi_{0}$ are the porosity and mass density corresponding to the reference pressure $p_{0}$, and $c_{f}$ is the isothermal compression coefficient of the fluid. $c_{\phi }$ is the pore compression coefficient.

Combine the above two formulas, then

8 $$ \rho \phi = \rho_{{0}} \phi_{0} [1 + (c_{f} + c_{\phi } )(p - p_{0} )] + o(c^{2} ) $$

where $o(c^{2} )$ represents a term with a compression coefficient of 2 or more. Assuming the comprehensive compression coefficient $c_{t} = c_{f} + c_{\phi }$, then

9 $$ \frac{\partial (\rho \phi )}{{\partial x}} = \rho_{0} \phi_{0} c_{t} \frac{\partial p}{{\partial t}} $$

By substituting Eq. (9) into Eq. (3), then

10 $$ \rho_{0} \phi_{0} c_{t} \frac{\partial p}{{\partial t}} = - \frac{\partial (\rho v)}{{\partial x}} $$

Therefore, the dynamic equations of one-dimensional non-Darcy seepage in fractured rock mass are obtained from Eqs. (5) and (10),

11 $$ \left\{ \begin{gathered} \rho_{0} \phi_{0} c_{t} \frac{\partial p}{{\partial t}} = - \frac{\partial (\rho v)}{{\partial x}} \hfill \\ \rho g_{a} \frac{\partial v}{{\partial t}} = - \frac{\partial p}{{\partial x}} - \frac{\mu }{k}\nu - f\nu^{2} - \rho g \hfill \\ \end{gathered} \right. $$

When the compressibility of water is not considered, it is obtained by Eq. (12):

12 $$ \left\{ \begin{gathered} \frac{\partial p}{{\partial t}} = - \frac{1}{{\phi_{0} c_{t} }}\frac{\partial v}{{\partial x}} \hfill \\ \frac{\partial v}{{\partial t}} = - \frac{1}{{\rho_{0} g_{a} }}[\frac{\partial p}{{\partial x}} + \frac{\mu }{k}\nu + f\nu^{2} + \rho_{0} g] \hfill \\ \end{gathered} \right. $$

Perform a dimensionless transformation of the above equation, let.

$$\overline{p} = \frac{p}{{p_{0} }}$$, $$\overline{x} = \frac{x}{H}$$, $$\overline{v} = \frac{v}{\mu /fk}$$, $$\overline{t} = \frac{t}{fkH/\mu }$$.

obtain

13 $$ \left\{ \begin{gathered} \frac{{\partial \overline{p}}}{{\partial \overline{t}}} = - n_{0} \frac{{\partial \overline{v}}}{{\partial \overline{x}}} \hfill \\ \frac{{\partial \overline{v}}}{{\partial \overline{t}}} = - n_{1} \frac{{\partial \overline{p}}}{{\partial \overline{x}}} - n_{2} \overline{\nu } - n_{3} \overline{\nu }^{2} - n_{4} \hfill \\ \end{gathered} \right. $$

**Figure 1:**
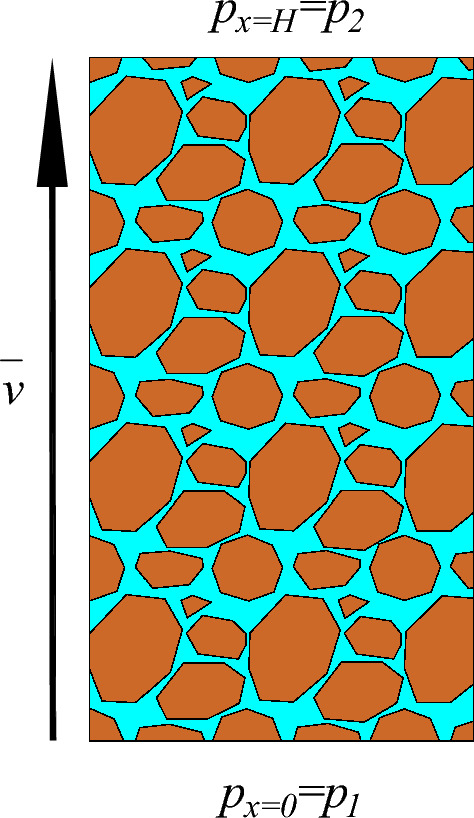
Schematic diagram of seepage from broken rock mass.

There into.

$$n_{0} = \frac{1}{{p_{0} \phi_{0} c_{t} }}$$, $$n_{1} = \frac{{p_{0} }}{{g_{a} \rho_{0} }}(\frac{fk}{\mu })^{2}$$, $$n_{2} = \frac{fH}{{g_{a} \rho_{0} }}$$, $$n_{3} = n_{2}$$, $$n_{4} = (\frac{fk}{\mu })^{2} \cdot \frac{H \cdot g}{{g_{a} }}$$;

Initial and boundary conditions of one-dimensional non-Darcy seepage system of fractured rock mass:

Schematic diagram of seepage from broken rock mass is shown in Fig. 1. Set the initial conditions of the seepage system: pore pressure $p_{0} (x) = p_{01} + \frac{{p_{02} - p_{01} }}{H}x$ (Where *H* is the accumulation height of the broken rock body, and $p_{01}$, $p_{02}$ are the initial pore water pressure at the lower and upper ends of the accumulated rock body, respectively), seepage velocity ${\text{v}}_{0} (x) = v_{0}$, seepage direction along the x-axis upward.

Boundary condition:$\left. p \right|_{x = 0} = p_{1}$, $\left. p \right|_{x = H} = p_{2}$, where the $p_{1} > p_{2}$, the flow direction is upward along the x-axis.

After nondimensionalization, we can get:$\overline{p}_{0} (\overline{x}) = \frac{{p_{01} }}{{p_{0} }} + \frac{{p_{02} - p_{01} }}{{p_{0} }}\overline{x}$, ${\overline{\text{v}}}_{0} (\overline{x}) = \frac{fk}{\mu }v_{0}$, here $\overline{x} \in \left[ {0,1} \right]$; $\left. p \right|_{x = 0} = \frac{{p_{1} }}{{p_{0} }}$, $\left. p \right|_{x = H} = \frac{{p_{2} }}{{p_{0} }}$.

### Equilibrium state of one-dimensional non-Darcy seepage system in fractured rock mass

The equilibrium state of the system $(\overline{p}_{s} ,\overline{v}_{s} )$ is found below, and when the system is in equilibrium, it is obtained by Eq. (14).

14 $$ \left\{ \begin{gathered} \frac{{\partial \overline{v}_{s} }}{{\partial \overline{x}}} = 0 \hfill \\ n_{1} \frac{{\partial \overline{p}_{s} }}{{\partial \overline{x}}} + n_{2} \overline{\nu }_{s} + n_{3} \overline{\nu }_{s}^{2} + n_{4} = 0 \hfill \\ \end{gathered} \right. $$

According to the pore pressure boundary condition of the percolation system of accumulative fractured rock mass is $\left. {\overline{p}_{1} } \right|_{{\overline{x} = 0}} = \frac{{p_{1} }}{{p_{0} }}$, $\left. {\overline{p}_{2} } \right|_{{\overline{x} = 1}} = \frac{{p_{2} }}{{p_{0} }}$, Then, pore water pressure $\overline{p}_{s}$ and seepage velocity $\overline{v}_{s}$ at equilibrium can be obtained respectively.

15 $$ \overline{p}_{s} (\overline{x}) = \frac{{p_{1} }}{{p_{0} }} + \frac{{p_{1} - p_{2} }}{{p_{0} }}\overline{x} $$

16 $$ \overline{v}_{s} = \frac{{ - n_{2} \pm \sqrt {n_{2}^{2} + 4n_{2} (n_{1} \frac{{p_{1} - p_{2} }}{{p_{0} }} - n_{4} )} }}{{2n_{2} }} $$

Because $$n_{1} = \frac{{p_{0} }}{{g_{a} \rho_{0} }}(\frac{fk}{\mu })^{2}$$, $$\frac{{p_{1} - p_{2} }}{{p_{0} }} > 0$$, $$n_{3} = n_{2} = \frac{fH}{{g_{a} \rho_{0} }}\left\{ \begin{gathered} > 0,{\text{ f}} > 0 \hfill \\ = 0,{\text{ f}} = 0 \hfill \\ < 0,{\text{ f}} < 0 \hfill \\ \end{gathered} \right.$$

So in Eq. (16). $n_{1} \frac{{p_{1} - p_{2} }}{{p_{0} }} - n_{4} = \frac{1}{{g_{a} \rho_{0} }}\left( {\frac{fk}{\mu }} \right)^{2} (p_{1} - p_{2} - \rho_{0} gH) > 0$.

That is, when $f > 0$, $n_{3} = n_{2} > 0$ the system has only one equilibrium state

$$ \overline{v}_{s} = \frac{{ - n_{2} \pm \sqrt {n_{2}^{2} + 4n_{2} (n_{1} \frac{{p_{1} - p_{2} }}{{p_{0} }} - n_{4} )} }}{{2n_{2} }} = \frac{{ - 1 \pm \sqrt {1 + \frac{4}{{n_{2} }}(n_{1} \frac{{p_{1} - p_{2} }}{{p_{0} }} - n_{4} )} }}{2} $$

When $f < 0$, $n_{3} = n_{2} < 0$, the equilibrium state of the system can be obtained from Eq. (16).

$$ \overline{v}_{s} = \left\{ {\begin{array}{*{20}c} { - \frac{1}{2} \mp \sqrt {1 + \frac{4}{{\left| {n_{2} } \right|}}(n_{1} \frac{{p_{1} - p_{2} }}{{p_{0} }} - n_{4} )} } & {1 + \frac{4}{{\left| {n_{2} } \right|}}(n_{1} \frac{{p_{1} - p_{2} }}{{p_{0} }} - n_{4} ) > 0} \\ { - \frac{1}{2}} & {1 + \frac{4}{{\left| {n_{2} } \right|}}(n_{1} \frac{{p_{1} - p_{2} }}{{p_{0} }} - n_{4} ) = 0} \\ \end{array} } \right. $$

If $f_{s} = - \frac{{\mu^{2} H}}{{4k^{2} (p_{1} - p_{2} - \rho_{0} gH)}}$, then all equilibrium states of the system are

17 $$ \overline{v}_{s} = \left\{ {\begin{array}{*{20}c} { - \frac{1}{2} + \frac{1}{2}\sqrt {1 - f/f_{s} } ,} & {{\text{f}} > 0} \\ { - \frac{1}{2} \mp \frac{1}{2}\sqrt {1 - f/f_{s} } ,} & {{\text{f}}_{s} < f < 0} \\ { - \frac{1}{2},} & {{\text{f}} = f} \\ \end{array} } \right. $$

**Figure 2:**
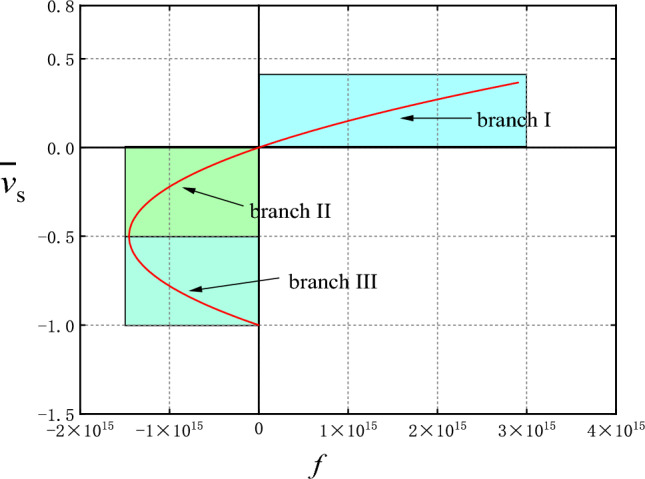
Solution of non-Darcy water seepage system in fractured rock mass.

All the solution results are drawn into the system solution diagram, as shown in Fig. 2. It can be seen that the equilibrium state of the system has three branches. Namely, branch I ($f > 0,\overline{v}_{s} > 0$), branch II ($f < 0,\overline{v}_{s} > - 0.5$), and branch III ($f < 0,\overline{v}_{s} < - 0.5$).

## Numerical simulation of nonlinear seepage characteristics system in fractured rock

The numerical calculation is carried out by the successive sub-relaxation iterative method^30^. According to Eq. (13), the forward difference formula is used for the partial derivative of time and the central difference formula is used for the partial derivative in the height direction. Dividing nodes along time and height directions respectively, with *i* representing time and *j* representing space, then there is

$$ \left\{ {\begin{array}{*{20}l} {\overline{p}_{i + 1,j} = \overline{p}_{i,j} + \Delta \overline{t} \cdot ( - n_{0} )\frac{{\overline{v}_{i,j + 1} - \overline{v}_{i,j + 1} }}{{2\Delta \overline{x}}}} \hfill \\ {\overline{v}_{i + 1,j} = \overline{v}_{i,j} + \Delta \overline{t} \cdot [ - n_{1} \frac{{\overline{p}_{i,j + 1} - \overline{p}_{i,j - 1} }}{{2\Delta \overline{x}}} - n_{2} \overline{v}^{2}_{i,j} - n_{4} ]} \hfill \\ \end{array} } \right. $$

Then, according to the sub-relaxation iterative formula $\overline{p}_{i + 1,j} \leftarrow (1 - \omega )\overline{p}_{i,j} + \omega \overline{p}_{i + 1,j}$, $\overline{v}_{i + 1,j} \leftarrow (1 - \omega )\overline{v}_{i,j} + \omega \overline{v}_{i + 1,j}$ (where $\omega$ is the relaxation factor, $0 < \omega < 1$). The time series of pore pressure and seepage velocity of each node can be obtained by iterative calculation of each point.

Set the pressure boundary $p_{1} = 0.65$ (MPa), $p_{2} = 0.25$ (MPa), fractured rock mass at reference pressure $p_{0} = 0.35$ (MPa), the initial porosity of fractured rock mass under reference pressure is $\phi_{0} = 0.3$, and the dynamic viscosity $\mu = 1.0 \times 10^{ - 3}$(Pa s), and the permeability $k = 0.5 \times 10^{ - 13}$ (m^2^), acceleration coefficient $g_{a} = 9.5 \times 10^{9}$, liquid compressibility coefficient $c_{f} = 4.77 \times 10^{ - 10}$ (Pa^−1^), the pore compressibility coefficient $c_{\phi } = 2.01 \times 10^{ - 9}$ (Pa^−1^), $\rho_{0} = 1000$ (kg/m^3^), *H* = 5m, $\Delta \overline{x} = 0.1$ (here the height of the accumulated rock mass is divided into 10 equal parts), $\omega = 0.5$.

### Branch I

The deviation factor of Darcy flow is taken $f = 3.5 \times 10^{12}$ (kg/m^4^), and the equilibrium state of the system $\overline{v}_{s} = 6.1{62} \times 10^{ - 4}$ can be obtained by calculation. If both pore water pressure and seepage velocity at the initial moment deviate from the equilibrium state relatively close, take $p_{01} = {0}{\text{.5}}$(MPa), $p_{02} = 0$(MPa), $\overline{v}_{0} = {6}{\text{.5}} \times 10^{ - 4}$.

**Figure 3:**
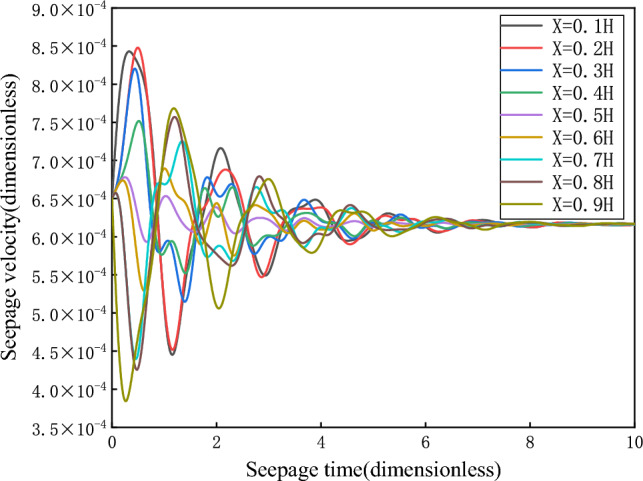
Seepage velocity change curve.

**Figure 4:**
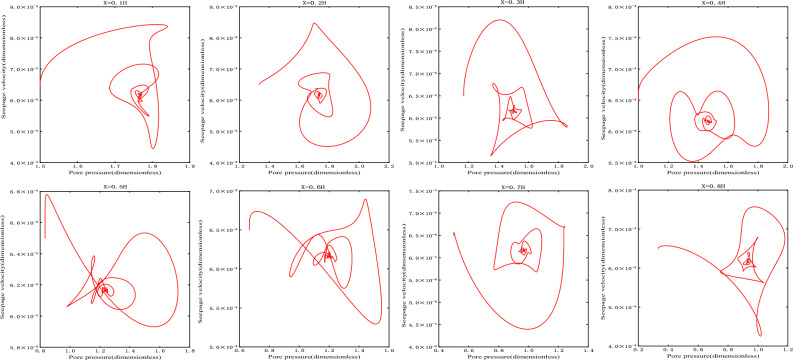
Phase orbit.

As shown in Fig. 3, the damping motion of the seepage velocity through oscillation attenuation approaches to the equilibrium state. The phase orbitals of different heights are shown in Fig. 4. At this time, the attractor is the stable focus in two-dimensional space, and its corresponding equilibrium state is stable.

**Figure 5:**
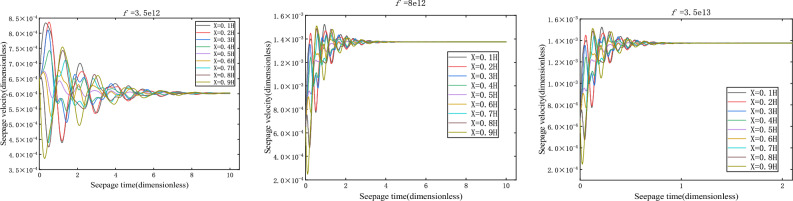
Seepage velocity change curve.

With the deviation of Darcy flow from factor *f*, how will the whole seepage system change. Keeping the boundary conditions and initial velocity constant, take $p_{01} = {0}{\text{.5}}$ (MPa), $p_{02} = 0$ (MPa), $\overline{v}_{0} = {6}{\text{.5}} \times 10^{ - 4}$; The values of *f* are $3.5 \times 10^{12}$ (kgm^−4^), $8 \times 10^{12}$ (kgm^−4^), and $3.5 \times 10^{13}$ (kgm^-4^), respectively. The relationship between seepage velocity and time is shown in Fig. 5.

In fact, for *f* greater than 0, no matter what value *v* takes, the system is stable and eventually converges to $v_{s}$. However, if the Darcy flow deviates from factor *f*, the system changes significantly. When *f* is greater than 0, as *f* decreases, the time required for the system to reach the equilibrium state becomes longer, and the time history curves of seepage velocity and pore pressure converge slowly. With the increase of *f*, the time required for the system to reach the equilibrium state becomes shorter, and the corresponding time history curves of seepage velocity and pore pressure converge faster. According to the above analysis, the equilibrium state corresponding to branch I is stable.

### Branch II

It can be seen from the bifurcation diagram that when the Darcy flow deviates from factor $f < 0$, there are two branches in the equilibrium state bounded by the limit point: branch II and branch III. Then there are two equilibrium velocities for each parameter *f*, denoted as $\overline{v}_{s2}$, $\overline{v}_{s3}$, respectively. Obtain a corresponding limiting parameter $f_{s}$ from the parameters given above,$f_{s} = - \frac{{\mu^{2} H}}{{4k^{2} (p_{1} - p_{2} - \rho_{0} gH)}}$. Therefore, when the parameter* f* changes between $f_{s} < f < 0$, the stability of the equilibrium state on each branch can be observed through the numerical calculation results. According to the parameters given above, the limit parameters are calculated $f_{s} = - 1.453133903133904 \times 10^{15}$ (kg/m^4^).

**Figure 6:**
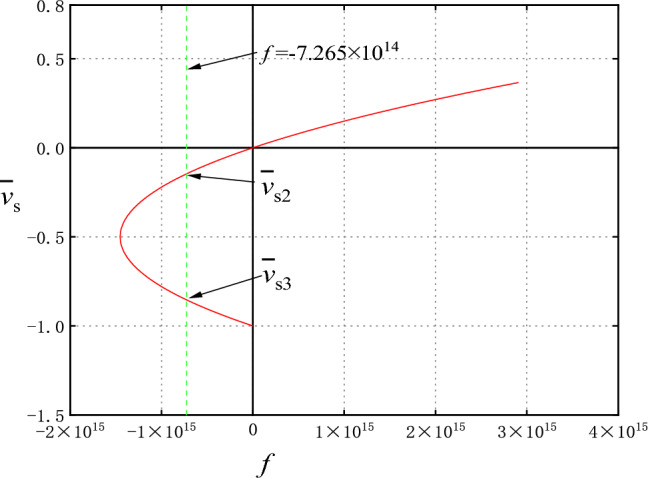
System Diagram.

As shown in Fig. 6, choose a *f* value between: $f_{s} < f < 0$, $f = - 7.265 \times 10^{14}$(kg/m^4^). And the corresponding velocity of the two equilibrium states on the solution graph are $\overline{v}_{s2} = - 0.1464$, $\overline{v}_{s3} = - 0.8536$, respectively.

**Figure 7:**
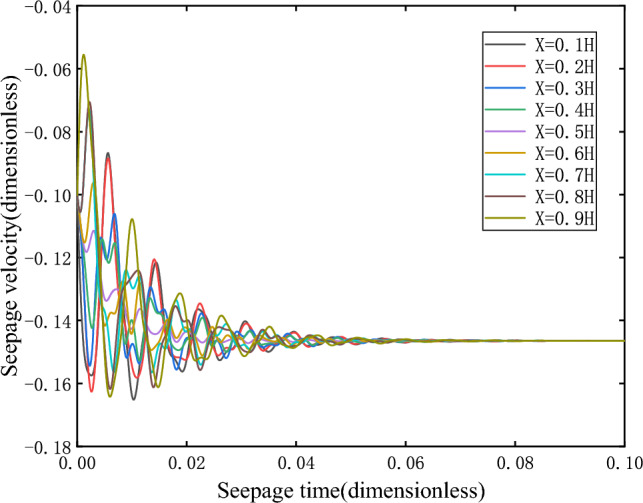
Seepage velocity change curve.

**Figure 8:**
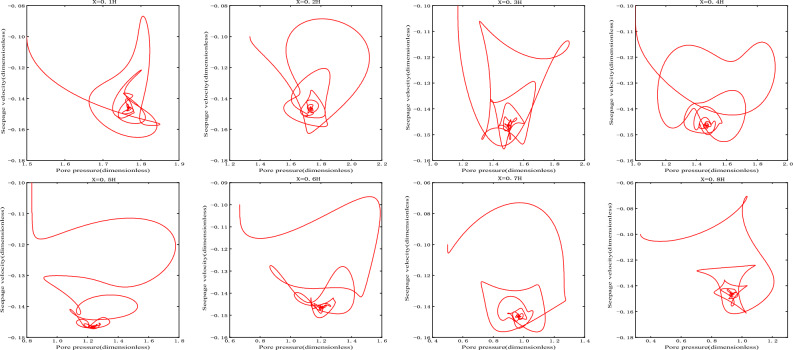
Phase orbit.

1. When $\overline{v}_{02} > \overline{v}_{s2}$, take $\overline{v}_{02} = - 0.1$, the initial velocity deviates from the equilibrium state and is located on the upper side of the equilibrium state, and finally returns to the equilibrium state $\overline{v}_{s2}$ stably after the evolution trajectory, as shown in the Fig. 7, which is the dynamic response when the initial velocity of branch II is greater than the equilibrium state velocity. Its phase trajectory at different heights is shown in Fig. 8, which converges to one point after a series of changes.

In particular, it should be noted that when $f < 0$, the time in the time history curve of all physical quantities such as pore pressure and seepage velocity is the size or absolute value of $\overline{t}$(the non-dimensional time).

**Figure 9:**
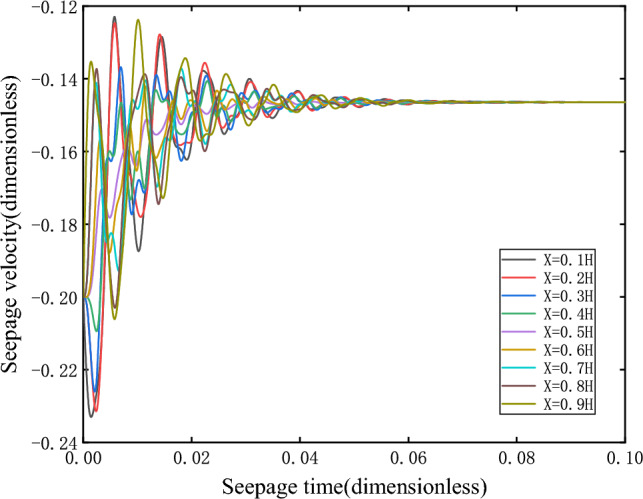
Seepage velocity change curve.

**Figure 10:**
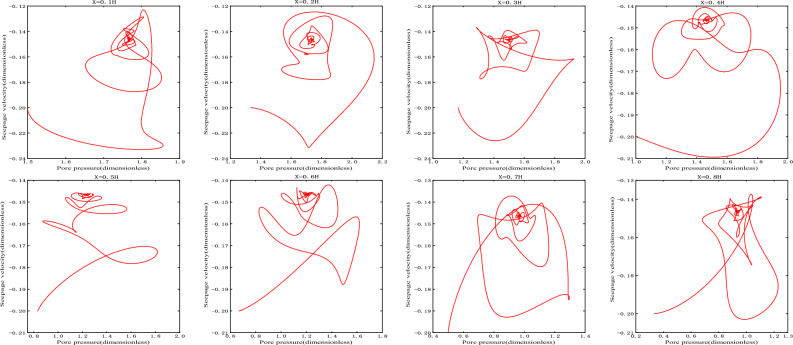
Phase orbit.

(2) When $\overline{v}_{02} < \overline{v}_{s2}$, take $\overline{v}_{02} = - 0.2$, the initial velocity deviates from the equilibrium state and is located on the lower side of the equilibrium state, and finally returns stably to the equilibrium state $\overline{v}_{s2}$(− 0.1464) after the evolution trajectory, as shown in the Fig. 9. As shown in Fig. 10, the phase trajectory composed of seepage velocity and pore pressure at different heights eventually converges to a point.

It can be seen that the equilibrium point corresponding to branch II is a stable node.

### Branch III

**Figure 11:**
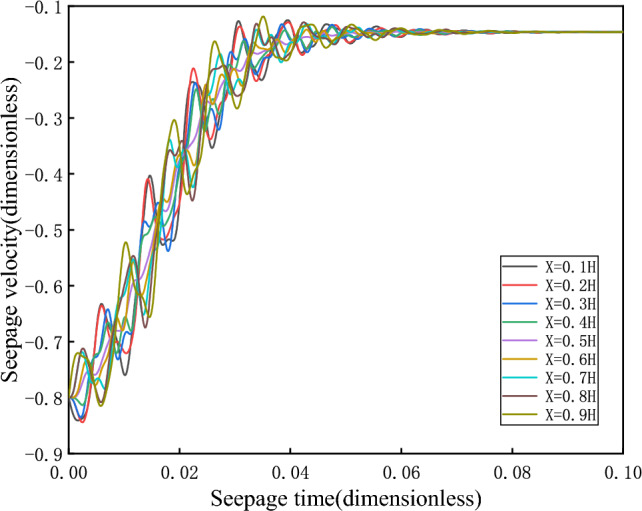
Seepage velocity change curve.

**Figure 12:**
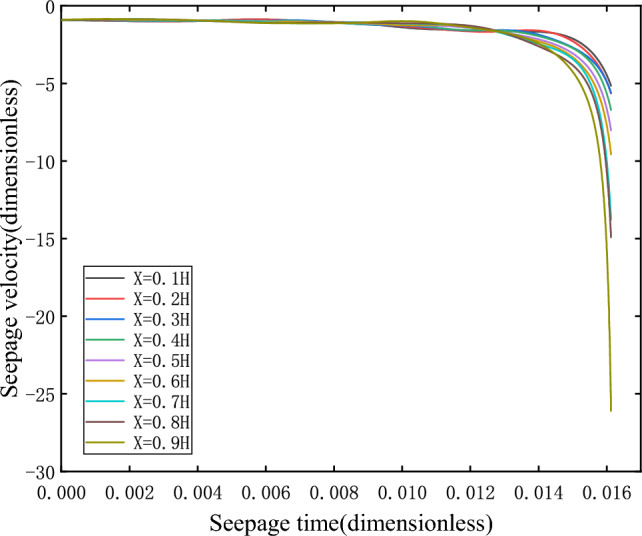
Seepage velocity change curve.

1. When $\overline{v}_{03} > \overline{v}_{s3}$, take $\overline{v}_{03} = - 0.8$, the initial velocity deviates from the equilibrium state and is located on the upper side of the equilibrium state. After evolution, it is found that the orbit does not return to its corresponding equilibrium state $\overline{v}_{s3}$, but is attracted to the corresponding equilibrium point $\overline{v}_{s2}$ on branch II, as shown in Fig. 11.
2. When $\overline{v}_{03} < \overline{v}_{s3}$, $\overline{v}_{s3} = - 0.8536$, take $\overline{v}_{03} = - 0.9$, the initial velocity deviates from the equilibrium state and is located on the lower side of the equilibrium state. After evolution, it is found that the orbit does not return to its corresponding equilibrium state $\overline{v}_{s3}$, but more and more deviates from the equilibrium state and flows to negative infinity, as shown in Fig. 12.

As can be seen from the changes in speed and pressure, the sudden change of instability when the system is unstable is extremely fast.

It can be seen that the equilibrium state corresponding to branch III is unstable. When the initial velocity is greater than the equilibrium state velocity, the orbit is eventually attracted to the corresponding equilibrium point on branch II. However, when the initial velocity is less than the equilibrium velocity, its orbit moves away from the equilibrium point and tend to negative infinity, that is, branch III is the boundary between the attraction domain of the stable node trajectory on branch II and the attraction domain of infinity. Therefore, the equilibrium point on branch III is the saddle point.

## Conclusions

The stability analysis of seepage system is the theoretical basis for correctly predicting and preventing fault water inrush disaster. A dynamic system is inevitably subject to various unpredictable perturbations. These disturbances can be small changes in the surrounding environment, such as fluctuations in air flow, temperature, or electromagnetic fields, or they can be intrinsic fluctuations in the system, such as the thermal motion of molecules and atoms. The stability of the solution (state of motion) means that if the system deviates from the solution (state of motion) under disturbance, it automatically returns to the state of motion represented by the solution. That is, the system can stay in this state of motion stably for a long time or at least not deviate too far from it. Otherwise, the solution of the equation is said to be unstable. Where Lyapunov stability means that the solution does not deviate too much under perturbations or small changes in initial conditions. In the asymptotically stable case, even if the system is disturbed, it eventually returns to the undisturbed solution (state of motion). In the case of instability, any disturbance or small change in the initial conditions is sufficient to cause the subsequent solution (state of motion) to deviate beyond any given range.

According to the non-Darcy characteristics of fractured rock seepage, the motion equation of fractured rock seepage is established in this paper. Combined with continuity equation and state equation, one-dimensional nonlinear dynamic partial differential equations for non-Darcy seepage in fractured rock mass are given. By means of dimensionless transformation, the equilibrium solution diagram of the seepage dynamic system under the given boundary conditions is obtained. With the change of parameter *f*, the equilibrium solution diagram of the seepage system can be divided into three branches. The numerical analysis shows that the equilibrium state (Branch I) is stable when the parameters $f > 0$ are used. When $f < 0$, the equilibrium state has two branches, the equilibrium point on one branch (Branch II) is a stable node, and the equilibrium point on the other branch (Branch III) is an unstable saddle point. When *f* gradually decreases and approaches the limit parameter $f_{s}$ (negative value), the saddle point meets the node and annihilates. When $f_{s} < f$ the equilibrium point disappears, the small disturbance of the system causes the seepage loss stability. Near the limit parameter, the stability of the equilibrium state of the system changes abruptly, so that the seepage system is prone to collapse mutation at the bifurcation point leading to water inrush and other disasters. The analysis in this paper can provide theoretical reference for the prediction of the water seepage instability in fractured rock mass.

## Data Availability

Some or all data, models, or codes generated or used during the study are available from the corresponding authors by request.
